# Could the Decision of Trial Participation Precede the Informed Consent Process? Evidence From Burkina Faso

**DOI:** 10.1371/journal.pone.0080800

**Published:** 2013-11-15

**Authors:** Lea Paré Toe, Raffaella M. Ravinetto, Susan Dierickx, Charlotte Gryseels, Halidou Tinto, Noèl Rouamba, Ibrahim Diallo, Yacouba Cissao, Korotimi Bayala, Susanna Hausmann, Joan Muela, Umberto D’Alessandro, Koen Peeters Grietens

**Affiliations:** 1 Institut de Recherche en Sciences de la Santé (IRSS)/Centre Muraz, Bobo-Dioulasso, Burkina Faso; 2 Department of Clinical Sciences, Institute of Tropical Medicine, Antwerp, Belgium; 3 Department of Pharmaceutical and Pharmacological Sciences, Catholic University (KU), Leuven, Belgium; 4 Department of Public Health, Institute of Tropical Medicine, Antwerp, Belgium; 5 Partners for Applied Social Sciences (PASS) International, Tessenderlo, Belgium; 6 Disease Control & Elimination Theme, Medical Research Council, Fajara, The Gambia; Nottingham University, United Kingdom

## Abstract

**Background:**

Over the last years, the number of clinical trials carried out in low-income countries with poor medical infrastructure and limited access to health care has increased. In these settings, the decision of participating in a clinical study may be influenced by factors related to participants’ vulnerability that limit the efficacy of the informed consent.

**Methods:**

A mixed methods social science study, based on the triangulation of qualitative and quantitative data, was carried out in a socio-economically disadvantaged and semi-urban area of Bobo Dioulasso, Burkina Faso. The study aimed at assessing the relevance of the informed consent procedure on the decision-making process of the parents and/or guardians of potential participants in a pediatric malaria trial.

**Results:**

For most parents (70.4%), the decision of participating had already been taken before undergoing the informed consent process and was based on the information conveyed through the community. Access to free and good quality health care often inspired this decision. In addition, the parents’ willingness to have their child included in the trial made them develop active strategies to achieve this purpose.

**Discussion:**

In a context of socio-economic vulnerability and poor access to free health care, the process of informed consent does not always accomplish its goal of informing people and enabling them to make a free and informed decision. This information role is somehow anticipated by the community and trial participation becomes a strategic action to secure otherwise unavailable health resources leading community members to decide on participation even prior to the informed consent process.

## Introduction

Over the last few years, the number of clinical trials conducted outside of the European Union [1] and the United States [2] has significantly increased. This phenomenon has also occurred in sub-Saharan Africa, partly because of the need for research addressing local health priorities [3] and thanks to increased funds for public health-oriented research. Clinical research is regulated by internationally agreed ethical principles [4-6] that have been translated into widely accepted methodological guidelines, national laws and regulations [7,8]. One of these principles, *respect for persons*, incorporates two ethical concepts [5], namely (i) individuals should be treated as autonomous entities, and (ii) persons with diminished autonomy should be protected. In medical research, the first concept implies that *competent* subjects must be free to decide whether or not to participate in research. It assumes that competent individuals are able to take an informed decision after the *informed consent* process, in which all relevant information is provided, discussed and understood. The concept of diminished autonomy refers to the fact that some individuals are not considered competent to consent, e.g. young children [9,10], minors [11–13] or individuals suffering specific physical/mental conditions [14,15]. In these cases, additional measures for their protection (i.e. the consent of a legal representative) must be taken. 

Nevertheless, the individual’s willingness and capacity of taking an unbiased decision on clinical trial participation may be hampered by other factors, one of them being ‘therapeutic misconception’, meaning that the distinction between medical care and medical research is misunderstood [16]. The individual’s capacity of taking an unbiased decision may also be limited in medical emergencies [17–19] or when an individual is desperately ill or has a terminal condition, as the enrolment in a clinical trial may represent the last hope to receive a life-saving treatment [20–22]. 

Diminished power in informed decision-making is not only linked to individual factors; it can also occur at group or community level. For instance, certain otherwise competent groups are potentially *vulnerable* to exploitation because of illiteracy [23], institutionalization (e.g. individuals that are part of highly hierarchical structures such as detainees and/or soldiers), and socio-economical marginalization or deprivation [24]. 

Though some factors limiting the efficacy of the informed consent process (such as therapeutic misconception) are ubiquitous, others are more frequent in low-income countries, such as high illiteracy rates and poor access to good medical care [25]. In this study the efficacy, strengths and limitations of the informed consent process, particularly the goal of *voluntariness*, were assessed in a clinical trial carried out in a low-income context. 

## Methods

### Study site and population

This social sciences study was carried out at the Dafra Health Centre in Bobo Dioulasso, Burkina Faso, among the parents of children screened and/or recruited in the clinical trial “In Vivo and in Vitro Efficacy of Antimalarial Treatments in Children in Burkina Faso” (MALACTRES), aiming at establishing the efficacy of the two national first-line antimalarial treatments. The study area is characterized by informal (i.e. without property titles), semi-urban settlements, with the furthest households situated approximately 5 km away from the health center. Economic activities are mostly informal and include small-scale trading and subsistence farming. Malaria is the most common cause of hospitalization and death [26]. Treatment seeking itineraries are mainly based on medical pluralism, meaning the exploration of a diversity of therapeutic pathways to recover health. For malaria, self-medication is often the first recourse. If symptoms persist, treatment is generally sought at the health centre. Patients and/or households bear all costs for any of these treatment choices, translating into a significant financial burden for the household. In fact, almost half of the population (42.8%) lives below the poverty line, with a daily income estimated at less than one$ [27]. The price of a malaria treatment (ASAQ) is around 0.6$ for an adult, 0.4$ for an adolescent and 0.3$ for a child, while the costs of attendance at the health facility are of 0.6$ for children and 1.5$ for adults.

### The Malactres trial

The Malactres clinical trial (ClinicalTrials.gov identifier: NCT00808951), to which this social science study was linked, was a phase IV, open label, parallel group study investigating the *in vivo* and *in vitro* efficacy of artemether-lumefantrine (AL) and amodiaquine-artesunate (ASAQ) in children with uncomplicated malaria. It was sponsored by the IRSS/Centre Muraz (Burkina Faso) and funded by the European Union (FP7). Children aged 6 months to < 15 years, with mono-infection with *P. falciparum* (parasitemia of 4,000–200,000 asexual parasites per µl), fever (axillary temperature ≥ 37.5 °C or fever in the last 24 hours) and whose parents/guardians agreed on participation, were eligible to participate. Exclusion criteria included body weight < 5 kg, haemoglobin < 5.0 g/dl, participation in any other investigational study during the previous 30 days, known hypersensitivity to study drugs, severe malaria, severe malnutrition, as well as other known intercurrent illness or condition which would place the subject at risk or interfere with the study results. The primary end-points were the unadjusted and adjusted treatment failure at day 42. The secondary end-points were unadjusted and adjusted treatment failure at day 28, fever clearance time, asexual parasite clearance time, gametocytaemia at day 7, 14, 21, 28, 35 and 42, and safety profiles. Randomisation numbers were computer-generated in blocks of 20. Overall, 440 children were recruited, randomized and treated with either AL or ASAQ, and actively followed up for 42 days. Recruitment was carried out during three periods: from December 2008 to February 2009, from July 2009 to March 2010, and from July to December 2010. Before the first recruitment period, information related to the trial had been given to the community inside the trial area by the research team.

### Study design, data collection and sampling

**Figure 1:**
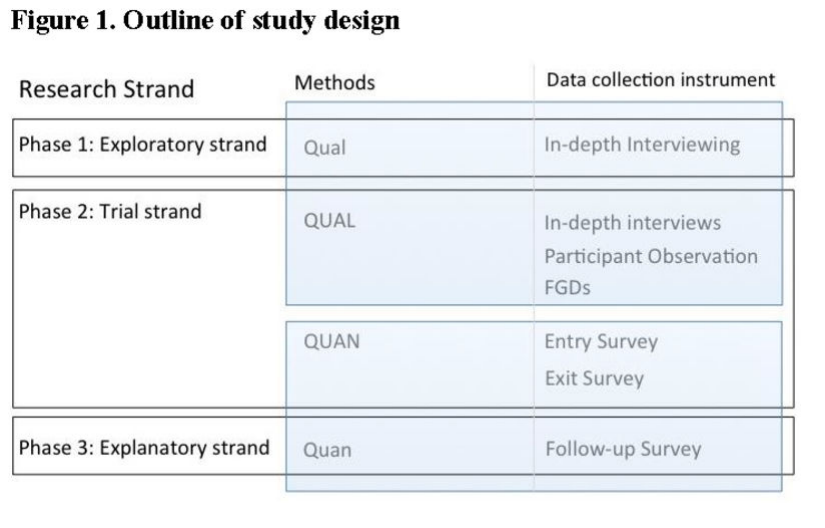
Outline of Study Design.

This study triangulated qualitative and quantitative methods and consisted of three strands, i.e. exploratory, trial and explanatory (in standard notation [qual → quan+qual → quan], as described in Figure 1. 

The Exploratory Strand consisted of qualitative research in the local communities. 

During the Trial Strand, the qualitative ethnographic data were triangulated with the quantitative data collected at the Dafra health center by means of an Entry and Exit Survey for the parents and/or guardians. 

During the Explanatory Strand, a questionnaire was administered to a random sample of the same parents and/or guardians. 

#### Exploratory qualitative strand

In order to explore possible relevant variables for the second strand of the study, two months before the start of the third period of recruitment for the Malactres trial, in-depth interviews were carried out with ten parents/guardians whose children had been previously included in the trial. Parents/guardians were purposefully sampled (i.e. participants are selected on purpose for the information they can provide and that could not be obtained as well from other choices). Informal interviews were also conducted with fifteen mothers, fathers or guardians whose children had not been screened for the trial but were aware the trial was ongoing. Informants were theoretically sampled (i.e. gradually selected in accordance with emerging results/theory) at community level. The interviews focused, among other factors, on knowledge/awareness of the Malactres trial, general motivations of community members for participating in the trial, and perceived possible advantages and risks related to the trial. The in-depth interviews with parents/guardians of recruited children were recorded and transcribed, while for parents/guardians of non-recruited children, notes were taken during or immediately after the informal interviews.

#### Trial Strand

The Trial Strand consisted of participant observation, focus groups discussions and quantitative surveys. 

Participant observation was carried out at the health centre on a daily basis during the third Malactres recruitment period and consisted of informal discussions with purposefully selected parents/guardians attending the health center with children younger than 15 years old. Informal interviewing was preferred in order to put the interviewees at ease and reduce the possible social desirability bias related to the place of the survey (i.e. health facility) and the type of questions (e.g., the perception of the trial, the research team and local health staff). The questions focused on the reasons for attendance, category of health providers planned to visit, i.e. health staff or research personnel, and on the knowledge of the trial. 

One focus group discussion (FGD) was conducted among purposefully selected parents/guardians whose children were screened but not included in the trial. The FGD aimed at obtaining additional information on the respondents’ opinions concerning the trial’s inclusion/exclusion criteria. The parents/guardians were approached after screening, when leaving the health center. Fourteen people participated in the discussion.

Two brief structured questionnaires were administered at the health center. The first one (Entry Survey) was administered to all parents/guardians arriving with a child at the health center. It consisted of six questions focusing on the reasons for attendance, awareness about the ongoing clinical trial and related expectations. 

For the second one (Exit Survey), parent-child pairs were approached at the time they were leaving the health center. Parents/guardians whose children had been screened for the trial were included. The survey consisted of eleven questions aiming at determining the level of understanding of the information conveyed during the informed consent process, the respondents’ reasons for participating, and their knowledge of the trial’s experimental nature, objectives, risks and benefits. 

#### Explanatory strand

A follow-up survey was carried out two months after the end of recruitment on randomly selected parents/guardians whose children had been screened and who had agreed to be re-contacted. They were administered a structured questionnaire (Follow-up Survey), in order to determine the remaining level of understanding of the trial objectives and procedures, the overall perception of the trial and the sources of information on its benefits and risks. 

### Data analysis

#### Qualitative data analysis

Data collection and analysis were concurrent and data analysis was a continuous, flexible and iterative process. Preliminary data were intermittently analyzed in the field after which further research was conducted confirming or refuting temporary results through constant validity checks. All transcriptions of recorded interviews and focus group discussions and the notes of informal conversations and observations were managed and analyzed in NVivo 9 (QSR International). 

#### Quantitative data analysis

All data were entered in Epidata 3.1 (www.epidata.dk) and analyzed with Stata 11 (www.stata.com). Frequencies were calculated for the main outcome variables. Fisher’s test was carried out to assess the role of prior knowledge of the trial on decision to participate. 

#### Data quality and bias

Given the sensitive nature of the questions, measures were taken to increase the quality of the survey data (in terms of wording and question order) as well as to avoid over-burdening the participants. Qualitative exploratory data had already shown that parents/guardians did not want to ‘lose time’ at the health center, especially when leaving. Therefore, socio-demographic information was collected only during the explanatory strand, within the community, and not at the health center. 

### Ethical considerations

The study was approved by the Ethics Committee of the Centre Muraz (ref.10-2010/CE-CM) and by the Institutional Review Board of the Institute of Tropical Medicine, Antwerp, Belgium (ref.10125715). Before fieldwork, local health and administrative authorities were informed about the study objectives and the field activities. Ethnographic research followed the Code of Ethics of the American Anthropological Association [28]. All interviewees gave oral informed consent after the explanation of the objectives and procedures in the local language. Oral consent was preferred and approved by the above-mentioned committees, since the interviewees were not put at risk of being harmed in their safety or psychological well-being. All parents/guardians who were proposed to participate gave their consent to do so. All original recordings, transcriptions and notes were made anonymous. Access to the database was restricted to the members of the research team. 

## Results

Overall, 560 parents/guardians participated in the Entry Survey. Four hundred and eight children met the trial pre-screening criteria and were asked to complete the Exit Survey (Trial Strand), 162 of which were recruited for the trial (Table 1). The Follow-up Survey included 112 households, 70 of which had at least one child included in the trial. 

**Table 1:** Overview Survey participants (Trial Strand).

**Phase 2. Trial Phase**	**N**	**Instrument**
**Caregivers of potential participants attending the Health Center**		
Caregivers of participants not screened by the research team	152	Entry Survey
Caregivers of participants screened by the research team	408	Entry + Exit Surveys
*Total*	*560*	
**Caregivers of participants screened by the research team**		
Caregivers with recruited child(ren)	162	Entry + Exit Surveys
Caregivers with non-recruited child(ren)	225	Entry + Exit Surveys
Caregivers unable or refusal to participate in the survey	9	Entry + Exit Surveys
Missing	12	Entry + Exit Surveys
*Total*	*408*	
**Phase 3. Explanatory Phase**		
**Screened caregivers surveyed in community**		
Caregivers with recruited child(ren)	70	Follow-up Survey
Caregivers with non-recruited child(ren)	42	Follow-up Survey
*Total*	*112*	

Almost all respondents (98.2%, 110/112) were married, mostly illiterate women (84%, 94/112 without formal schooling). Their mean age was 32.7 years (+/- 7.8 SD) (Table 2). 

**Table 2:** Socio-demographic information of the surveyed in the community (Explanatory Strand).

**Mean age (years)**	**Range**	**SD**
32.7	20-55	7.8
**Socio-demographic information respondents (Total= 112)**	**Frequency**	**Percentage**
**Place of residence**		
Informal settlement	111	99,1
Other neighbourhoods	1	0,9
**Gender**		
Female	110	98,2
Male	2	1,79
**Level of schooling**		
No schooling	94	83,9
Primary	12	10,7
Secondary	6	5,4
**Marital status**		
Married	110	98,2
Not married	2	1,8
**Person accompanying child to the health center**		
Mother	102	91, 1
Father	1	0,9
Guardian	9	8

### Awareness of the trial upon arrival at health centre

Upon arrival, more than half (62.7%, 351/560) of the interviewed parents/guardians were already aware of the ongoing trial (Entry Survey, Table 3). Indeed, 59.7% (334/560) of them were attending the health centre because of the research team (referred to as the Centre Muraz team and the ‘people that bring aid’). This proportion was higher (70.4%, 114/162) in the subgroup of parents/guardians of children that would be later recruited in the trial (Table 4, Exit Survey).

**Table 3:** Decision making on trial participation of all caregivers of children attending the health centre (Trial Strand).

**Overview from the Entry Survey**	**Frequency**	**Percentage**
**Reason for attending the health centre**		
To consult the health centre staff	214	38,2
To consult the team of the Centre Muraz	160	28,6
To consult ‘the people that bring aid’	174	31,1
Don’t know	1	0,18
Other	11	1,97
*Total*	*560*	*100*
**Awareness of Malactres Trial**		
People attending health centre aware of the trial	351	62,7
*Decision to participate upon arrival at the health centre*		
Already taken	342	97,4
Not yet decided	4	1,1
Depends	5	1,4
*Subtotal*	*351*	*100*
- People attending health centre not aware of the trial	208	37,1
*Intention to participate if asked*		
Would participate	178	85,6
Would not participate	17	8,2
Have not decided yet	7	3,4
Do not know	6	2,9
*Subtotal*	*208*	*100*
Missing	1	0,2
*Total*	*560*	*100*
**Participants seeking out the trial for participation**		
*Participants arriving to see Centre Muraz staff; and aware of The Malactres Trial, and with decision taken to participate*	350	62,5

**Table 4:** Decision making on trial participation of caregivers of children recruited in the trial (Trial Strand).

**Reasons for participation and awareness of the trial**	**Frequency**	**Percentage**
**Reason for attending the health centre**		
To consult the health centre staff	44	27,1
To consult the team of the Centre Muraz	57	35,2
To consult ‘the people that bring aid’	57	35,2
Don’t know	4	2,5
*Total*	*162*	*100*
**Awareness of Malactres Trial**		
People attending health centre aware of the trial	118	73
*Decision to participate upon arrival at the health centre*		
Already taken	117	99,1
Not yet decided	1	0,9
Depends	0	0
*Subtotal*	*118*	*100*
People attending health centre not aware of the trial	44	27,0
*Intention to participate if asked*		
Would participate	41	93,0
Would not participate	3	7,0
*Subtotal*	*44*	*100*
*Total*	*162*	*100*
**Participants seeking out the trial for participation**		
*Participants (i) arriving to see Centre Muraz staff; and, (ii) aware of The Malactres Trial, and (iii) with decision taken to participate in trial.*	114	70,4

Both the qualitative and quantitative data confirmed that the information parents/guardians had received on the trial before meeting the study team came largely from friends and family. Only 14.3% (16/112) of parents whose children were screened stated to have heard about the trial for the first time from the health staff. The content of the information received at community level centered mainly on ‘free health care’ and ‘people working on malaria’ and ‘helping children’ (Table 5).

**Table 5:** Descriptive analysis of the Follow-up Survey (Explanatory Strand).

**Sources and content of the information leading to decision making**	**Frequency**	**Percentage**
**Reported source of initial information on the trial**		
Neighbors	67	59,8
Health staff	16	14,3
Relatives	5	4,5
Other	13	11,6
Missing	11	9,8
*Total*	*112*	*100*
**Reported content of the information initially provided**		
People providing free care	39	34,8
People working on malaria	31	27,7
People helping children	25	22,3
Clinical trial with medicines	1	0,9
Taxi money as benefit	3	2,7
Missing	13	11,6
*Total*	*112*	*100*

### Decision-making

As showed in Table 3, among those parents/guardians aware of the on-going study upon arrival at the health centre, the large majority (97.4%, 342/351) had already decided, before the informed consent procedure, to participate in the study if the opportunity were offered. Among parents/guardian who were unaware of the study when arriving at the health center, a similarly high proportion (85.6%, 178/208) stated they would participate if any such study was available. More than half (62.5%) of all parents/guardians was (i) already aware of the ongoing trial; and (ii) claimed to have attended the health facility to seek out the research team; and, at the same time, stated (iii) they had already decided to participate in the trial. We did the Fisher’s exact test on the role of prior knowledge of the trial on decision making and the difference was significant (P<0,001). For (iii), the most common reasons for participation, as determined in the Exit Survey, were the perceived ‘aid’ provided by the trial (67.3%), the better quality of care (31.5%) and the better quality of the medication (13.0%) provided (Table 6). 

**Table 6:** Descriptive analysis of Exit Survey response (Trial Strand).

Overview of the Exit Survey after the informed consent process	Frequency	Percentage
**Reasons to have accepted to participate in the trial** (multiple responses)		
Aid provided by the trial	109	67,3
Better quality of care	51	31,5
Better quality of the medication	21	13,0
Other	3	1,9
**Knowledge of the reasons behind the study**		
Yes	8	4,9
No	154	95,1
*Total*	*162*	*100*
**Knowledge of the benefits of the trials**		
Able to mention trial benefits	158	97,5
Unable to mention trial benefits	4	2,5
*Total*	*162*	*100*
**Knowledge of the possible risks for the child**		
Yes	4	2,6
No	152	97,4
Missing	6	
*Total*	*162*	*100*
**Knowledge of the difference in medication for the children in the study**		
Yes	82	50,6
No	41	25,3
Don’t know	39	24,1
*Total*	*162*	*100*
**Knowledge of the possible discomforts of the medication**		
Yes	17	10,5
No	115	71,0
Don’t know	30	18,5
*Total*	*162*	*100*
**Knowledge of the possible negative effects of the medication**		
Yes	10	6,2
No	105	64,8
Don’t know	47	29,0
*Total*	*162*	*100*
**The trial team mentioned that the personal information is confidential**		
Yes	1	0,6
No	161	99,4
*Total*	*162*	*100*

### Perception of the trial in the community

The local communities knew there was a team working on malaria, perceived to offer aid and free–of-charge treatment. The research team was often referred to as “les gens qui aident” (i.e. people providing help) or identified as the ‘Centre Muraz’s people’, as opposed to the health providers at the health facility where the trial was carried out. Receiving help for their children was the main motivation for agreeing to participate in the trial. This aid referred to the free-of-charge health care and, in a few cases (2.7%), to the ‘taxi money’ (*l’argent du taxi*) provided as compensation for transport costs. Similarly, in the follow-up survey, the large majority of respondents mentioned provision of aid (57.1%) and improvement of children’s health (21.4%) as the aim of the trial, while only 10% mentioned that the study compared two different treatments (Table 7).

**Table 7:** Descriptive analysis of the Follow-up Survey responses of caregivers of children recruited in the trial (Trial Strand).

**Overview topics from the Follow-up Survey**	**Frequency**	**Percentage**
**Aim of the trial according to participants**		
To compare different medication	7	10,0
To improve the child’s health	15	21,4
Providing aid	40	57,1
Do not know	8	11,4
*Total*	*70*	*100*
**Children experiencing problems during trial**		
Yes	0	0
No	70	100
*Total*	*70*	*100*
**Awareness of possibility of leaving trial**		
Yes	2	3
No	68	97
*Total*	*70*	*100*
**Considering drop-out during the trial**		
Yes	0	0
No	70	100
*Total*	*70*	*100*
**Person signing the informed consent form**		
Mother	63	90,0
Father	3	4,3
Other	3	4,3
Missing	1	1,4
*Total*	*70*	*100*
**Literacy**		
Illiterate (not able to read)	9	12,9
Literate (able to read)	60	85,7
Missing	1	1,4
*Total*	*70*	*100*
**Obtaining more information on consent from relatives**		
Yes	6	8,6
No	64	91,4
*Total*	*70*	*100*
**Still have the informed consent form**		
Yes	60	85,7
No	10	14,3
*Total*	*70*	*100*
**Received money from research team**		
Yes	70	100
No	0	0
*Total*	*70*	*100*
**Use of ‘taxi money’**		
Food	63	90,0
Article of clothing	15	21,4
Transport	1	1,4
Care	1	1,4
*Total*	*70*	*100*
**Perception of Centre Muraz** (multiple options)		
Institution providing aid	50	71,4
Institution providing health care	13	18,6
Institution providing free-of-charge health care	5	7,1
Research institution	2	2,9
*Total*	*70*	*100*
**Perception of Centre Muraz** (forced choice)		
Institution providing aid	65	92,9
Institution providing health care	67	95,7
Institution providing free-of-charge health care	59	84,3
Research institution	21	30,0
*Total*	*70*	*100*

Both in the exploratory and trial strands, health care provided by the research team was perceived as being of better quality than the one usually available. Taking more time for the clinical examination, performing diagnostic tests prior to treatment and providing medication of better quality were among the reasons given for agreeing to participate in the trial (Table 8, section 3, Q8-9).

**Table 8:** Quotes illustrating qualitative findings (Exploratory and Trial Strands).

**1. Key Information conveyed during informed consent process**
Q1. This is research study is being done to learn more about the treatment of malaria. We are carrying out a research study to compare different medicines for the treatment of mild malaria (Malactres Patient Information Sheet).
Q2. With this study we want to find out their efficacy and safety and also see if he parasite is resistant to them (Malactres Patient Information Sheet).
Q3. The study medicine that your child will receive will be determined by a process of randomization. Randomization means that your child will receive by a study nurse one of the 2 medicines studied by chance (Malactres Patient Information Sheet).
Q4. Your child’s participation in this study is completely voluntary. If you decide that you do not want to participate in the study or decide to withdraw your child from the study at any time and for any reason, this will not affect your child’s care at the outpatient department, where standard care for all medical problems is available. During the study, you will be informed promptly of any new information that may influence your willingness to continue participation in the study.
Q5. Should you decide to withdraw your child from the study before your child has finished the course of study medicines, then your child will receive the local standard treatment for malaria from the study team, but after the standard treatment has been given, medical care will no longer be provided by the study team. If the child is withdrawn from the study after completion of the course of study medicines, then no further care will be provided by the study team (Malactres Patient Information Sheet).
**2. Research as Aid**
Q6. People know we are poor, so they help us’ [Interview participating mother]
Q7. The doctors know that we do not have the money to treat our children so they treat them free of charge’. [Interview participating mother]
**3. REPORTED REASONS FOR TRIAL PARTICIPATION**
Q8. The doctors of the Centre Muraz take the child’s blood and check if he really has malaria while the others only ask what is wrong with the child’ [Informal conversation with participating mother].
Q9. When the other doctors give my children medication, two days later the disease starts again. That’s why I wanted the team of Muraz to treat him” [Informal conversation with participating mother].
**4. Strategies for trial enrolment**
Q10. When I got to the health centre, I didn’t know I shouldn’t have told the doctors that I had already given some medication to my child. Had I known that they refused to enrol my child for that I would never have told them” [Interview with mother of a non-recruited child].
**5. Perception of the trial in The community**
Q11. I had received information on the work from women who came from the health centre with their children. They told me that someone was treating children free-of-charge’ [FGD. Mother of a non-recruited child]
Q12. I heard women say that there were people at the health centre who treat children free-of-charge, so I went’ [Informal conversation with mothers of recruited children].

### Comprehension of research procedures and therapeutic misconception

#### Aid

Despite the explicit mention of ‘research’ in the informed consent process (Table 8, Q1), the qualitative analysis of narratives shows that the distinction between aid and research was often not understood by the respondents and that people considered the provision of aid, seen as a logical consequence of their poverty, as the main feature of the trial (Table 8, section 2, Q6-7). 

#### Randomization

Despite having the concept of randomization explained during the informed consent process (Table 8, section 1, Q3), only 25.3% (41/162) of respondents had retained that all children did not receive the same medication during the trial (i.e. two different types of medication were given to different groups of children). The qualitative research revealed that even among those who had retained the information about the use of the two different treatments, it was believed that the allocation of a specific treatment was the consequence of the children’s different constitutions and/or health problems. 

#### Risks and benefits

During the Exit Survey, only a few (2.6%, 4/162) parents/guardians stated that there were risks related to trial participation (Table 6). Conversely, the large majority (97.5%, 158/162) was able to state trial benefits. However, only few (10.5%) affirmed that the trial could cause discomfort to the child or that the medication could have side effects (6.2%). 

#### Confidentiality

Only 1 person (1/162) (Table 6) had retained any information about the right to confidentiality, despite the fact that this was explained in the informed consent process.

#### Withdrawing from the study

Two respondents (2/162) (Table 6) had retained they could withdraw from the study at any time (Table 8, section 1, Q4-5). 

### Informal enrolment strategies

Qualitative data from the Exploratory and Trial Strands showed that community members were not passive participants, rather active actors in the trial enrolment process, to the extent of developing strategies to increase the chances of their child being included in the trial. These strategies included providing erroneous information to the study team, e.g. not declaring the treatment taken by the child before attendance or not reporting non-malaria symptoms such as coughing or colds or other complications. Other strategies consisted in attending the health center very early in the morning; or, situating oneself in a clearly visible position in the waiting room to be more easily noticed by the trial staff (as some days not all potential patients could be screened); or sending someone else to assure such a place in the waiting room; and, trying to negotiate inclusion through an acquaintance of a member of the trial team. 

The large majority (86%, 60/70) of parents/guardians of recruited children carefully stored their signed informed consent form, even after the end of trial, as evidence of trial participation (Table 7). This is *per se* good practice, but proving previous trial participation was generally believed to give the former participants a higher chance of enrolment in future trials or to allow them to benefit from free-of-charge routine health care after the trial.

## Discussion

The process of informed consent translates the ethical principle of respect for persons into practice and is founded on three main principles, namely information, comprehension and voluntariness [5]. Patients and/or their parents or guardians should be provided with clear, complete and balanced information on the proposed clinical trial so that they can freely decide on their participation. Failing to do so would open the door to potential abuse and malpractice. Nevertheless, besides the information received during the consent process, other factors can influence the patient’s or parent’s decision. Clearly, in the low-income setting where this study was carried out, most parents were aware of the ongoing trial and wanted their child to be enrolled before having received any information from the research team; rather, based on the information circulating in their community. This means that the decision to participate in the trial *preceded* the informed consent process, and was not necessarily dependent on the type and quality of the information provided by the study team, with the risk of biasing the consent process. This does not necessarily imply that the information provided during the informed consent process was inconsequential in influencing decision-making as it could have helped the parents confirm their pre-existing intentions. It is, nevertheless, clear that such decisions were often guided by additional factors and motives unrelated to the trial’s purposes, e.g. free and better quality health care and reimbursement of travel expenses. 

Another element potentially biasing the informed consent procedure was the lack of understanding of trial procedures among potential participants or their parents/guardians. Only a small percentage of parents retained key aspects of the trial procedures (i.e. main research aim, randomization, and the right to confidentiality) even when the questions were asked immediately after the informed consent procedure. It is possible that the apparent lack of understanding could be less of a problem of comprehension than of voluntariness or free decision-making, as it can be partially due to participants’ lack of interest in understanding the trial procedures in relation to the offered and/or perceived benefits. In addition, the clinical trial was a low-risk study as the study treatments corresponded to the recommended first line treatments, possibly explaining the little concern parents/guardians had over the potential risks. Parents’/guardians’ attitude towards a trial with a potentially higher risk, e.g. a phase II trial, may be different. 

In Burkina Faso, user-fees are required for malaria diagnosis and treatment, with no waiver for special categories of patients such as children under 5 years of age or pregnant women. Accordingly, malaria treatment can constitute a considerable financial strain for low-income households, especially during the malaria season. The provision of free-of-charge care as well as the accurate follow-up offered to the child after initial treatment made study participation attractive, to the point that 70,4% of the parents of recruited children had actively tried to have their child included in the trial. These factors imply a different way of interpreting trial participants’ role, namely as people *acting* upon the availability of the trial and not as *passive* participants being guided through an informed consent process. However, while practices such as early morning attendance to the health centre or trying to negotiate inclusion through an acquaintance in the clinical trial team would not increase the individual risk, providing erroneous information on previous symptoms, including those of severe malaria, may do so. The question remains whether the informed consent process in this setting fully achieves its objectives. 

The common and understandable wish of potential trial participants to be recruited in a clinical trial to benefit of the service offered, particularly for the socio-economically disadvantaged households, leads to a need for improved strategies to make the informed consent process more effective. This, however, is more easily said than done as the research team provided extensive information (in the local language) on the research and answered any questions the parents/guardians may have asked. 

The reputation of/trust in the research team and the perception of aid probably played a major role in decision-making. It is obvious that if the population had not trusted the research team, the recruitment and follow-up rate would have been much lower than observed. Nevertheless, the desire to secure otherwise unavailable good quality health care should not overshadow the fact that children were included in a research project, and not in a humanitarian program, even despite the low risk of the trial. 

In conclusion, in a context characterized by socio-economical vulnerability and poor access to free health care, the process of informed consent does not always accomplish its goal of enabling potential study participants to make a free and informed decision. This information role is somehow anticipated by the community and trial participation may become a strategic action to secure otherwise unavailable health resources leading community members to decide on participation even prior to the informed consent process. Given the direct benefits of trial participation and possible trust in researchers, innovative strategies to ensure a free decision-making process are required. 

## Supporting Information

Ethics S1 **Ethical approval of the Institutional Review Board of the Institute of Tropical Medicine, Antwerp, Belgium Faso.**
(PDF)

Ethics S2 **Ethical approval of the Ethics Committee of Centre Muraz, Bobo Dioulasso, Burkina.**
(PDF)

Ethics S3 **Informed consent leaflet and form of the MALACTRES trial (English version).**
(DOCX)

Ethics S4 **Informed consent leaflet and form of the social sciences study (in French).**
(DOCX)
