# Supplementary material for: Could the Decision of Trial Participation Precede the Informed Consent Process? Evidence From Burkina Faso
Source: PLoS One. 2013 Nov 15;8(11):e80800. doi: 10.1371/journal.pone.0080800 (PMC3829938; doi:10.1371/journal.pone.0080800)
Supplement: Ethics S1 — Ethical approval of the Institutional Review Board of the Institute of Tropical Medicine, Antwerp, Belgium Faso. (PDF) [file pone.0080800.s001.pdf]

Prins Leopold Instituut voor Tropische Geneeskunde
Institut de Médecine Tropicale Prince Léopold
Prince Leopold Institute of Tropical Medicine
Instituto de Medicina Tropical Príncipe Leopoldo
Stichting van openbaar nut | BE-410.057.701

Dr. A. Erhart
Department of Parasitology

IRB/AB/ac/089 8 June 2011

Dear Colleagues,

Re: **Mixed-methods study on clinical trial participation and the informed consent process in vulnerable populations**

Our ref: **11 21 5 774**

I am pleased to inform you that the IRB has approved the protocol of the above mentioned study during its meeting of 7 June.

The protocol does not need to be submitted to the EC of UZA.

Kind regards,

Prof. Anne Buvé
Chairperson IRB a.i

Anne Buvé - Chairperson Institutional Review Board
Institute of Tropical Medicine Antwerp
Nationalestraat 155, 2000 Antwerpen - Belgium
Tel: +32 3 247 65 33 abuve@itg.be

Ann Caron - Secretariat Institutional Review Board
Institute of Tropical Medicine Antwerp
Nationalestraat 155, 2000 Antwerpen - Belgium
Tel: +32 3 247 07 28 acaron@itg.be

**IRB number :**	**11 21 5 774**
**Title project/study:**	**Mixed-methods study on clinical trial participation and the informed consent process in vulnerable populations**
**Investigator:**	**Dr. A. Erhart**<br>**Departement Parasitologie**
Background and study objectives (rationale and relevance):	No problem.
Approach for the recruitment of the study subjects:	Not detailed for this quantitative part.
Study costs:	
Study procedures:	Sampling not provided.
Risk evaluation:	OK
Benefit evaluation:	OK
Confidentiality and privacy:	OK
Informed consent:<br>- language<br>- description of the content of the study<br>- foreseeable risks & benefits<br>- confidentiality<br>- privacy<br>- medical treatment<br>- contact information<br>- voluntary participation & refusal<br>- legal representative	 Ok<br>Ok<br>Ok<br>Ok<br>NA<br>OK<br>OK<br>NA
Final remarks:	
**Conclusion**	**Favourable (= IRB approval). The study does not need to be submitted to the EC of UZA.**

Anne Buvé – Chairperson Institutional Review Board
Institute of Tropical Medicine Antwerp
Nationalestraat 155, 2000 Antwerpen - Belgium
Tel: +32 3 247 65 33 abuve@itg.be

Ann Caron – Secretariat Institutional Review Board
Institute of Tropical Medicine Antwerp
Nationalestraat 155, 2000 Antwerpen - Belgium
Tel: +32 3 247 07 28 acaron@itg.be
