# Supplementary material for: Could the Decision of Trial Participation Precede the Informed Consent Process? Evidence From Burkina Faso
Source: PLoS One. 2013 Nov 15;8(11):e80800. doi: 10.1371/journal.pone.0080800 (PMC3829938; doi:10.1371/journal.pone.0080800)
Supplement: Ethics S2 — Ethical approval of the Ethics Committee of Centre Muraz, Bobo Dioulasso, Burkina. (PDF) [file pone.0080800.s002.pdf]

MINISTERE DE LA SANTE

SECRETARIAT GENERAL

Comité D'Ethique

BURKINA FASO

Unité – Progrès – Justice

N/Réf. A10-2010/CE-CM

Bobo-Dioulasso, le 23 mars 2010

Avis d'examen d'un Projet de Recherche

1. Numéro d'enregistrement du projet de recherche :
10-2010/CE-CM

2. Intitulé du Projet de recherche :
« Etude sur le processus du consentement éclairé dans un contexte de vulnérabilité des populations »
Titre en Anglais : « Multi-methods study on clinical trial participation and the informed consent process in vulnerable populations »

3. Investigateur Principal
Mme PARE/TOE Léa

4. Avis du Comité d'Ethique
Avis favorable du Comité d'Ethique à l'exécution du projet mais sous réserve de la prise en compte des amendements figurant sur le rapport de la réunion.

5. Durée de validité :
1 an à partir de la date d'approbation

6. Liste des membres présents :

1. **Dr. Germain TRAORE**
2. **Dr. Rasmané BEOGO**
3. **Dr. ZINGUE Dezemon**
4. **Mme ZAMPA Hato Odile**
5. **Mr SANFO Dramane**

Pour Le Président P/O

Dr. ZINGUE Dézemon

Comité d'Ethique Institutionnel du Centre MURAZ, BP 390 Bobo-Dioulasso, Burkina Faso, Tél. : (226) 20.97.01.02 Fax : (226) 20.97.04.57
