# Supplementary material for: Could the Decision of Trial Participation Precede the Informed Consent Process? Evidence From Burkina Faso
Source: PLoS One. 2013 Nov 15;8(11):e80800. doi: 10.1371/journal.pone.0080800 (PMC3829938; doi:10.1371/journal.pone.0080800)
Supplement: Ethics S3 — Informed consent leaflet and form of the MALACTRES trial (English version). (DOCX) [file pone.0080800.s003.docx]

**RESEARCH PARTICIPANT INFORMED CONSENT FORM**

**STUDY TITLE**

***In vivo* and *in vitro* efficacy of the recommended first line antimalarial treatments**

**(artemether-lumefantrine and amodiaquine-artesunate) in children with uncomplicated**

**malaria in Burkina Faso**

Please read the background information and informed consent form carefully. The background information explains your rights and our responsibilities to you. If you have any questions concerning the study please do not hesitate to ask any of the doctors. Before you decide, it is important for you to understand why the research is being done and what it will involve. You will be given a copy of this signed document (Informed Consent Form)to take home with you.

**YOU MUST KEEP THIS BACKGROUND INFORMATION WITH YOU THROUGHOUT THE STUDY PERIOD.**

**PURPOSE OF THE STUDY**

This research study is being done to learn more about the treatment of malaria. We are carrying out a research study to compare different medicines for the treatment of mild malaria. The medicines we are studying are: arthemeter-lumefantrine and amodiaquine-artesunate. Both drugs are active against malaria and are recommended by the Burkina Ministry of Health. With this study we want to find out their efficacy and safety and also see if the parasite is resistant to them.

**HOW THE STUDY IS DONE**

The child under your care will be treated for malaria with one of the above study medicines. After the treatment, your child will be actively followed for 42 days to see if the malaria infection is completely cured. If your child is not completely cured by the study medicines, s/he will then be given treatment according to the standard practice in your country. The study medicine that your child will receive will be determined by a process of randomization. Randomization means that your child will receive by a study nurse one of the 2 medicines studied by chance. You are being asked to allow your child (child under your care in the case of a legal guardian), to participate in this study. Your child will be actively followed up for 42 days or until such time as you or the study doctors decide that your child should no longer participate in the study. You can choose to withdraw your consent to participate in the study any time and without influencing the medical attention your child may need. Moreover, a blood sample (5ml) will be collected before the child is treated to test if the parasites are sensitive to different drugs. This will be repeated if your child present again with an infection during the follow up.

**PROCEDURES**

1) The study doctors will examine your child today.

2) A blood sample will be collected. A small amount of blood will be taken by fingerprick to examine for malaria parasites, to store blood samples on filter paper for future laboratory tests that will not impact on the health care of your child. An additional blood sample will be collected to be tested in the laboratory to see if the parasites are sensitive to a number of drugs.

3) If the diagnosis of malaria is confirmed, and your child is eligible for the study, treatment with either arthemeter + lumefantrine (A+L) or amodiaquine-artesunate. (AQ+AS) will be given by a study nurse or any other research team member at the clinic during the first 3 days.

4) You will be asked to return to the clinic at least 6 more times over the next month so that the success of the treatment can be judged. At each of the follow-up visits, your child will be examined by the study doctors and, a small amount of blood will be taken by fingerprick to examine for malaria parasites and to save on filter paper. If your child has again the disease, an additional blood sample will be collected to be tested in the laboratory to see if the parasites are sensitive to a number of drugs, like before starting the treatment.

5) In case of missing appointment, the home health visitor will visit your child at your home to find out why you missed the appointment and bring your child to the clinic for assessment.

6) If, at any time, the treatment given to your child does not seem to be working well, it will be changed to another treatment, according to the usual standard of care.

7) There will be someone at the study clinic every day from 8:00 am to 5:00 pm and at night. You can come to the clinic for evaluation anytime that your child is ill during the next 42 days.

8) Blood samples will be collected from an arm vein or from a finger by an experienced nurse. Blood sampling may cause pain and swelling. In addition small violet spots around the site of injection called hematoma could appear.

**RISKS AND DISCOMFORTS**

1) Side effects following treatment with the study medications could occur. Generally, side effects (nausea, headache, dizziness…) are expected to be mild and short-lived. Your child will be monitored closely after receiving treatment for malaria with the study medications for any possible side effects of the drugs and will receive appropriate medical care for any problem that happens during the course of the study.

2) Randomization: Your child will be assigned to a treatment group by chance. The drug will be administered by the study nurse or another research team member not involved in your child’ clinical assessment, and the treating physician will not know which of the two medicines has been given to your child, unless he/she thinks that it is better to know it for giving a better clinical care. The treatment your child receives may prove to be less effective or to have more side effects than the other study treatments or than other available treatments. This will not be known until after the study is completed.

3) Severe malaria: Your child may develop malaria that is severe even after receiving treatment with study medications. If your child shows any evidence of severe malaria (including persistent vomiting, low blood (anaemia), convulsions, confusion, or coma) treatment with the usual standard of care will be given and your child will be referred for possible admission to hospital.

4) Blood draws: The risks of drawing blood from a fingerprick include temporary discomfort from the needle stick, bruising, skin infection, and fainting. Venous blood will be drawn from the arm to see if the parasites are sensitive to several drugs. This will be done before treatment and if your child comes back with parasites in the blood. Each time the amount of blood drawn is equivalent to one teaspoon. The amount of blood removed will be too small to affect your child’s health.

5) Unknown Risks: The research treatments may have side effects that no one knows about yet. The researchers will let you know if they learn anything that might make you change your mind about your child’s participation in the study.

6) Confidentiality: Participation in research may involve a loss of privacy, but information about your child will be handled as confidentially as possible. Medical information related to malaria will be collected on your child and only people taking care of the child and/or the study personnel will have access to this information. Records will be kept as confidential as possible. You will also have the right to request and see the information collected during this study on your child.

**BENEFITS**

1) Your child will receive clinical care from the medical officers and nurses of the project staff in the study clinic. This will include care for unscheduled sick visits. 2) The knowledge gained from this study will help your country in determining the best treatment for uncomplicated malaria.

**COST/PAYMENT**

After enrolment in the study, you will not be charged for clinic visits or treatment. Your child will not be paid for participation in the study. We will reimburse any transport costs incurred for clinic visits and meals will be provided when your child is admitted for observation.

**ALTERNATIVES TO PARTICIPATION**

Your child’s participation in this study is completely voluntary. If you decide that you do not want to participate in the study or decide to withdraw your child from the study at any time and for any reason, this will not affect your child’s care at the outpatient department, where standard care for all medical problems is available. During the study, you will be informed promptly of any new information that may influence your willingness to continue participation in the study.

**CONSEQUENCES OF WITHDRAWAL**

Should you decide to withdraw your child from the study before your child has finished the course of study medicines, then your child will receive the local standard treatment for malaria from the study team, but after the standard treatment has been given, medical care will no longer be provided by the study team. If the child is withdrawn from the study after completion of the course of study medicines, then no further care will be provided by the study team.

**USE OF THE RESULTS**

The findings from this study may be published in a medical journal. The study participants will not be identified by name. After the study is completed, you may request an explanation of the study results.

**TREATMENT AND COMPENSATION FOR INJURY**

If you are injured or have questions about injuries as a result of being in the study, please contact the doctors in the study clinic. The services at the public health facility will be open to you in case of any such injury.

**VOLUNTARY PARTICIPATION**

Participation in this study is entirely voluntary. You have the right to refuse your child’s participation or to withdraw at any point in this study without negative consequences or loss of benefits to which you and your child are otherwise entitled.

**Implication of your SIGNATURE OR THUMBPRINT**

If you give consent for your child to participate in this study, you should sign or place your thumbprint in the consent form. Your signature or thumbprint below means that you understand the information given to you about your child’s participation in the study and in the consent form. You will also be asked to sign another copy of this informed consent form for documentation.

**CONSENT FORM FOR PARTICIPATION IN RESEARCH PROJECTS AND CLINICAL TRIALS**

**Study Title**

***In vivo* and *in vitro* efficacy of the recommended first line antimalarial treatments**

**(artemether-lumefantrine and amodiaquine-artesunate) in children with uncomplicated**

**malaria in Burkina Faso**

Local Investigator: ________________________________________________________

Address:___________________________________________________________________

Contact number_____________________________________________________________

I, …………………………………. mother/father/legal representative declare that I have

understood the objectives and purposes of this study. I agree that my child……………………………………………………………. participates in this study. I am aware that I can withdraw my child from the study at any time without any consequence

to my child or to me.

Name of parent/legal representative

Signature or Thumbprint of parent/ legal representative Date/Time
