# Supplementary material for: Could the Decision of Trial Participation Precede the Informed Consent Process? Evidence From Burkina Faso
Source: PLoS One. 2013 Nov 15;8(11):e80800. doi: 10.1371/journal.pone.0080800 (PMC3829938; doi:10.1371/journal.pone.0080800)
Supplement: Ethics S4 — Informed consent leaflet and form of the social sciences study (in French). (DOCX) [file pone.0080800.s004.docx]

**FICHE D’INFORMATION ET DE CONSENTEMENT ECLAIRE**

**Titre du projet**

**La participation aux essais cliniques et le processus de consentement éclairé dans un contexte de vulnérabilité des populations**

**Fiche d’information**

**But de l’étude**

Je (nom de l’enquêteur) viens de L’Institut de Recherche en Sciences de la Santé (IRSS) et je veux vous inviter à prendre part à une étude sur « le consentement éclairé ». Le consentement éclairé c’est lorsque les gens vous expliquent une étude et vous demandent votre accord avant de vous faire participer à l’étude. Vous avez participé ou vous avez entendu parlé de l’étude qui est réalisée au centre de santé de Dafra sur les médicaments utilisés pour soigner le paludisme. Les docteurs, normalement, avant de faire participer quelqu’un à l’étude expliquent le travail à la personne et lui demandent son accord. Nous voulons aujourd’hui faire une étude pour connaître votre opinion sur cette manière de travailler des docteurs c’est-à-dire la manière dont ils vous expliquent les objectifs de l’étude sur les médicaments du paludisme, les avantages et les inconvénients liés à votre participation à cette étude. Nous voulons que vous nous nous disiez ce que vous savez sur cette étude des médicaments du paludisme ainsi que les raisons qui vous amènent à participer à cette étude. Nous voulons également que vous nous dites ce qui vous dérange dans cette étude. Mais avant de participer à notre présente enquête, nous allons d’abord vous expliquer le travail et demander votre accord.

**Contenu de l’étude**

La présente étude consiste à vous poser des questions pendant environ trente minutes sur l’étude sur les médicaments du paludisme qui se réalisent au centre de santé de Dafra et sur la manière dont les docteurs vous demandent votre accord de participation.

**Confidentialité**

Vos réponses seront confidentielles c’est-à-dire qu’elles ne seront pas montrées aux docteurs ou à toute autre personne qui travaillent au centre de santé. Nous n’allons pas non plus écrire vos noms à côté de vos réponses. Les réponses seront connues seulement par les gens qui travaillent avec nous dans le cadre de ce travail. Toutes les fois où nous devons reporter les résultats de l’enquête, vos noms ne seront pas associés à vos réponses.

**Participation volontaire**

Votre participation à cette étude est entièrement volontaire et vous pouvez refuser a tout moment de répondre aux questions qui vous sont posées.

**Inconforts liés à la participation de l’étude**

La participation à l’étude va vous prendre un peu de votre temps, 30 mm environ. Mais vous allez vous-même choisir le moment et le lieu de l’enquête en fonction de votre disponibilité de temps.

**Bénéfices liés à la participation de l’étude**

Vous n’avez pas de bénéfice direct lié à votre participation a cette étude telle que la compensation financière. Mais les bénéfices issus de votre accord de participation sont collectifs c’est-à-dire qu’ils profitent à toute la communauté. Car vos réponses vont servir à améliorer la façon dont ces études sur le médicament se font dans les dispensaires et ça c’est à l’avantage de tout le monde.

**Personne à contacter en cas de besoin**

La présente étude a reçu l’approbation du comité d’éthique institutionnel du Centre Muraz. Pour toute question relative au projet, vous pouvez contacter le comité d’éthique du Centre Muraz ou Mme Léa PARE (70759361).

**FICHE D’INFORMATION ET DE CONSENTEMENT ECLAIRE**

**Titre du projet**

**La participation aux essais cliniques et le processus de consentement éclairé dans un contexte de vulnérabilité des populations**

**Fiche de consentement**

Je reconnais avoir pris connaissance des informations énoncées dans le formulaire des informations concernant l’étude ci-dessus nommée. J’ai eu la possibilité de poser des questions sur cette étude et j’ai obtenu des réponses satisfaisantes. Je comprends les conditions de participation et les inconforts et bénéfices liés à ma participation à cette étude.

J’ai compris que ma participation est volontaire et que je peux décider à tout moment de ne plus répondre aux questions de cette étude sans aucune pénalité.

Je soussigné (é) déclare donner volontairement mon accord pour participer à cette étude.

Non et prénom du participant___________________________________________________

Signature/emprunte digitale du participant_______________________________________

Nom et prénom de la personne administrant le consentement___________________________

Signature de la personne administrant le consentement_______________________________

Date et Lieu
